# Integrating knowledge and omics to decipher mechanisms via large‐scale models of signaling networks

**DOI:** 10.15252/msb.202211036

**Published:** 2022-07-26

**Authors:** Martin Garrido‐Rodriguez, Katharina Zirngibl, Olga Ivanova, Sebastian Lobentanzer, Julio Saez‐Rodriguez

**Affiliations:** ^1^ Heidelberg University, Faculty of Medicine, and Heidelberg University Hospital Institute for Computational Biomedicine, Bioquant Heidelberg Germany

**Keywords:** biological networks, cellular signaling, functional analysis, phosphoproteomics, transcriptomics, Computational Biology, Signal Transduction

## Abstract

Signal transduction governs cellular behavior, and its dysregulation often leads to human disease. To understand this process, we can use network models based on prior knowledge, where nodes represent biomolecules, usually proteins, and edges indicate interactions between them. Several computational methods combine untargeted omics data with prior knowledge to estimate the state of signaling networks in specific biological scenarios. Here, we review, compare, and classify recent network approaches according to their characteristics in terms of input omics data, prior knowledge and underlying methodologies. We highlight existing challenges in the field, such as the general lack of ground truth and the limitations of prior knowledge. We also point out new omics developments that may have a profound impact, such as single‐cell proteomics or large‐scale profiling of protein conformational changes. We provide both an introduction for interested users seeking strategies to study cell signaling on a large scale and an update for seasoned modelers.

## Graphical abstract

Network models based on prior knowledge are used to understand signal transduction. This Review compares and classifies recent network approaches according to their characteristics in terms of input omics data, prior knowledge, and underlying methodologies.

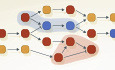

## Introduction

**Figure 1:**
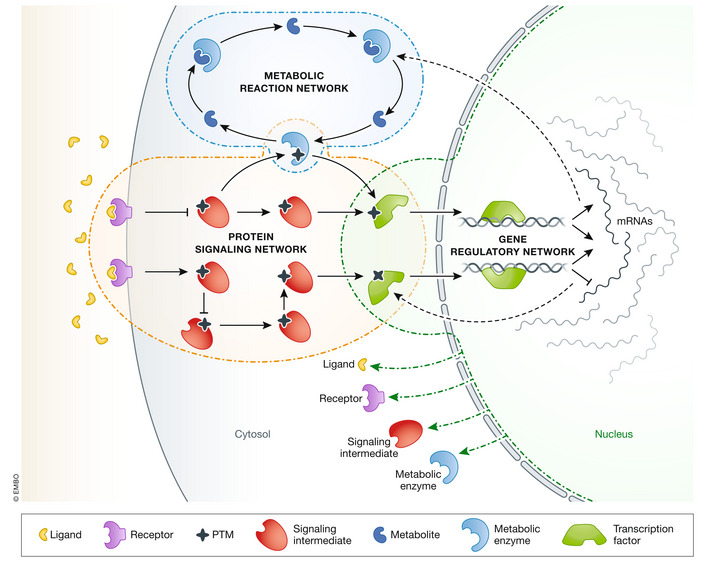
Three major types of intracellular biological networks: metabolic reaction networks, gene regulatory networks, and, the focus of this Review, protein signaling networks.

The cell senses, integrates, and transmits information from/to its environment via signaling cascades, largely mediated by proteins and their post‐translational modifications (Deribe *et al*, 2010). The activation or inhibition of these cascades usually results in new transcriptional and metabolic programs that depend on the received stimuli and can lead to different cellular responses (Weidemüller *et al*, 2021). In biology, networks are used to conceptually represent not only signaling, but also gene regulation and metabolism (see Fig 1). In these networks, nodes constitute biological entities (e.g., proteins or metabolites) and edges indicate known or predicted relationships between them. When networks are constructed based on previous discoveries, they are often referred to as prior knowledge networks (PKNs).

**Figure 2:**
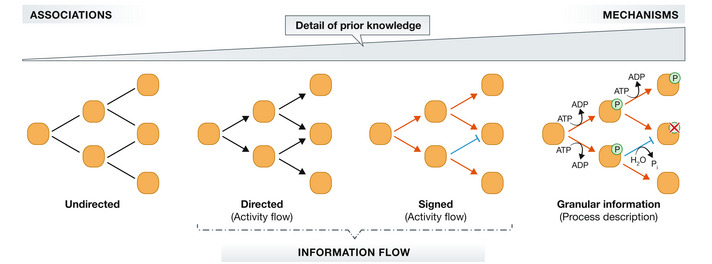
Prior knowledge networks may contain different levels of detail. Edge color and shape represents the sign of interactions: Activations (red) and inhibitions (blue).

Biological databases store and update PKNs that summarize decades of research and knowledge with variable levels of detail. While resources such as STRING (Szklarczyk *et al*, 2021) store undirected interactions, others like BioGRID (Oughtred *et al*, 2021) or SIGNOR (Licata *et al*, 2020) contain directed ones. The direction of the interaction usually reflects causal statements, while undirected edges describe other types of relationships or unspecific/unknown interactions (e.g., proteins bind, but it is unclear if this has a functional impact) (Touré *et al*, 2020). This level of detail can be further increased to answer different questions. The SBGN standard (Le Novère *et al*, 2009) defines (i) *Activity Flow* networks, representing biological processes as directed interactions among bioentities and (ii) *Process Description* networks, containing granular and detailed information, such as the subcellular location of proteins or reactions' kinetics and cofactors (Vogt *et al*, 2013). In general, the greater the amount of information contained in a PKN, the greater the amount of mechanistic knowledge that can be extracted from it, although this is also associated with a greater complexity (Fig 2).

PKNs can represent one or multiple types of biological processes. For instance, networks derived from the kinase‐substrate dataset in PhosphoSitePlus (Hornbeck *et al*, 2015) encode only a particular type, phosphorylation, while the PKN developed in COSMOS (Dugourd *et al*, 2021) is composed of reactions among metabolites, activations/inhibitions among signaling proteins and metabolite‐protein interactions. When a subnetwork is associated with an individual biological process, or is built around a particular biological entity, it is commonly referred to as a biological *pathway* **.** For example, focusing on a particular protein, a pathway may represent upstream and downstream regulatory processes associated with it (e.g., the TP53 pathway). Similarly, if we focus on metabolites, the term pathway is employed to refer to the set of reactions involved in their synthesis and/or consumption (e.g., the glycolysis pathway). Well‐known repositories of biological pathways are KEGG (Kanehisa *et al*, 2021), Reactome (Gillespie *et al*, 2022) and WikiPathways (Martens *et al*, 2021). Of note, nodes and interactions can be part of multiple pathways. For example, the TP53 transcription factor may appear both in the TP53 pathway and in the cell cycle pathway. The concept of pathways is commonly used to study signaling and is explicitly considered in some modeling methods.

PKNs can be combined with various types of molecular measurements to study the process that they represent in specific biological scenarios, such as disease. In this context, untargeted omics technologies have become increasingly popular, as they can be applied without defining a subset of interesting molecular features beforehand (Sobsey *et al*, 2020). Next‐generation sequencing (NGS) transcriptomics and mass‐spectrometry (MS) phosphoproteomics are common techniques to generate hypotheses about cellular signaling, and have been extensively applied in large consortia like The Cancer Genome Atlas Consortium (TCGA) (Cancer Genome Atlas Research Network *et al*, 2013) or the Clinical Proteomics Tumor Analysis Consortium (CPTAC) (Ellis *et al*, 2013). Phosphoproteomics provides measurements of phosphorylation events, a key mediator of signal transduction (Aebersold & Mann, 2016), while transcriptomic techniques measure the abundance of different types of RNA molecules (Stark *et al*, 2019), which are often used as a proxy for protein signaling activities (Szalai & Saez‐Rodriguez, 2020). Although these technologies offer comprehensive molecular measurements of the biological system under study, they are still far from covering all the axes of complexity that characterize cellular signaling (see Box 1).

Box 1 **The complexity of cellular signaling networks**

Cell signaling is a biological process that the scientific community has been trying to model and understand for decades, and there are several axes of complexity that complicate this effort. The methods examined in this Review abstract part of this complexity to scale the models to hundreds/thousands of molecules and large networks, relying on certain assumptions that we attempt to briefly decompose in this box.

First, signaling is a highly dynamic and interconnected biological process. At the structural protein level, events occur on a microsecond scale. Most phosphorylation cascades occur on a scale of seconds or minutes (Blazek *et al*, 2015). In contrast, the abundance of proteins is functionally regulated in a time scale of hours to days (Buccitelli & Selbach, 2020). Also, most signaling processes do not occur in a linear fashion and are instead affected and controlled by feedback mechanisms that create complex dynamic behaviors (Brandman & Meyer, 2008). Multiple computational models aim to capture the dynamics of signaling using methods like ordinary differential equations (Hass *et al*, 2019). However, these models need to estimate a large number of parameters from the data, as prior knowledge does not typically provide the kinetic details of most signaling reactions. Quantitative dynamic models of signaling become intractable for large networks as the search space of the parameters increases exponentially with the model size.

To simplify the modeling task, almost all methods described in this Review make the strong assumption of steady states of signal transduction and then compare these hypothetical steady states between measured biological conditions. In general, this assumption does not hold true, especially in response to perturbation, a scenario that is particularly informative of signaling processes (Saez‐Rodriguez & Blüthgen, 2020). It can, nevertheless, be a suitable assumption to model changes in basal signaling processes (e.g., tumor cells versus normal cells).

Cell signaling is intrinsically a spatial process. At the protein level, post‐translational modifications induce conformational changes that are essential for regulating protein activity (McClendon *et al*, 2014). When multiple proteins are considered, certain signal transmission events, such as phosphorylations, require physical contact between the entities involved. At a larger scale, some signaling phenomena consist of translocation between subcellular compartments, such as transcription factors that are activated and subsequently migrate to the nucleus (Weidemüller *et al*, 2021). Finally, signaling can also propagate between adjacent cells through juxta‐ and paracrine signaling (Gerosa *et al*, 2020). Similarly to temporal complexity, the lack of data limits the application and validation of the few computational methods that aim to capture signaling with spatial resolution.

In addition to time and space, there are many other elements that are simplified or disregarded when scaling up models of signaling. Examples include protein complex formation or priming phosphorylation events, both of which are required for certain signaling events to take place (Aoki & Yoshida, 2017). Moreover, when multiple interactions influence a single downstream protein, determining its signaling activity is not always possible from a simple graphical description (activity flow). While formalisms such as logic gates can deal with these situations (Abou‐Jaoudé *et al*, 2016), the information to define these gates is not always available, though they can be built by further manual curation or fitting to data.

Overall, cell signaling is a very complex process that we need to simplify to generate models that are able to digest a large number of measurements and vast networks.

It is also important to note that signaling PKNs, including pathways, are subject to certain levels of research bias. Previous work suggests that a small number of proteins have been more extensively studied and are better understood and easier to link to functions compared to others (Edwards *et al*, 2011; Weidemüller *et al*, 2021; Kustatscher *et al*, 2022). Related to this, signaling has been studied much more in certain contexts, such as cancer, than in other biological contexts (de Magalhães, 2021) (see Box 2).

Box 2 **Publication bias in prior knowledge**

To illustrate some of the biases in signaling prior knowledge, we retrieved data from OmniPath (Türei *et al*, 2016) and PubTator (Wei *et al*, 2019) as of 25‐04‐2022. We first created a phosphorylation‐driven signaling network from OmniPath. To do so, we restricted the “enzsub” collection, which comprises interactions between enzymes and substrates, to “phosphorylation” and “dephosphorylation” events. Then, we filtered out self‐interactions and interactions without at least one supporting reference (identified in OmniPath as those with a curation effort = 0). We used PubTator to match articles in PubMed to biological entities such as genes, cell lines, and diseases. In the signaling network, most nodes have a low degree (defined as the total number of incoming and outgoing interactions), while a small proportion of nodes account for the majority of interactions (Box Fig 1A). The degree distribution is partially correlated with the total number of times that nodes are mentioned in the PubMed database (Box Fig 1B). Some well‐known examples, such as TP53 or AKT1, are among the best studied and most connected nodes. We also investigated the most frequent entities found within the 12,170 articles providing bibliographic support for the signaling network (Box Fig 1C and Box Fig 1D). “HeLa” is the entity with the most mentions within cell lines, and the disease entity “Neoplasms” appears in 60% of the literature. These results illustrate how signaling interactions are investigated more in certain biological contexts than in others. Data and code to reproduce the results shown in the Box Figure have been deposited in https://zenodo.org/ with the DOI identifier 10.5281/zenodo.6541931.

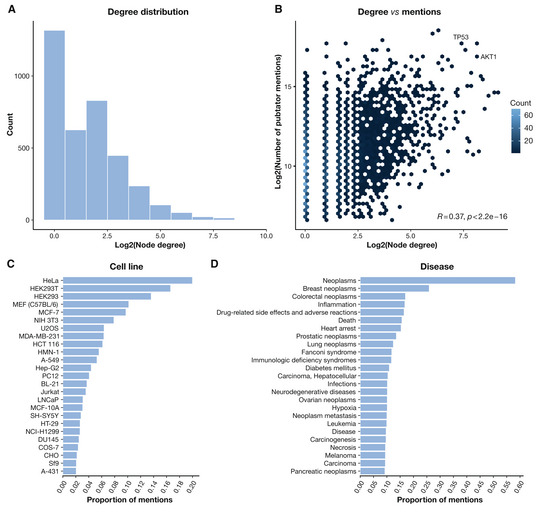

Biases in prior knowledge. (A) Histogram showing the node degree distribution in the signaling network (binwidth = 1). (B) Hexbin plot of node degree (X‐axis) versus number of mentions in PubTator (Y‐axis) for all the genes in the signaling network. The hexagon color represents the number of genes on each binned region (bins = 50). Bottom‐right text indicates the Pearson correlation coefficient between the two variables and its significance. The position of “TP53” and “AKT1” genes in the hexbin plot is labeled. (C, D) Barplots reflecting the top 25 biological entities with the highest proportion of mentions within the literature supporting the signaling network.

Bearing these limitations in mind, several computational methods have been developed in recent years to model cellular signaling through the integration of omics data and PKNs. Here, we review methods that:

1 Have an associated manuscript published in the last 10 years (2012–2022).
2 Have an open‐source software implementation.
3 Use measurements from transcriptomics, phosphoproteomics, or both as input.
4 Use directed networks (signed or unsigned) to represent prior knowledge.
5 Can handle input PKNs that contain more than 1,000 nodes (either as a whole or as a combination of multiple pathways).
6 Use the directionality of the PKN to some extent.
7 Output cellular signaling networks, or ranking of networks, that are expected to provide a mechanistic explanation for the observed patterns in the input omics data.

In total, we reviewed 35 methods and selected 14 fulfilling these criteria (Table 1.).

**Table 1:** List of the 14 methods that fulfilled our selection criteria. The table details the year of publication of the associated manuscript, the article URL, the implementation URL, and the type of implementation.

Method	Year	Article URL	Source code URL	Implementation	License
DEGraph	2012	URL	URL	R package	GPL‐3
CLIPPER	2013	URL	URL	R package	GPL‐3
TEAK	2013	URL	URL	MATLAB based software with GUI	GPL‐3
DEAP	2013	URL	URL	Python and R scripts	GPL‐3
TieDIE	2013	URL	URL	Python scripts	GPL‐3
sub‐SPIA	2015	URL	URL	R code	MIT
PHONEMES	2015	URL	URL	R package + external solver	GPL‐3
CausalR	2017	URL	URL	R package	GPL‐2
HiPathia	2017	URL	URL	R package	GPL‐2
TPS	2018	URL	URL	Scala, Python and Bash scripts	MIT
CARNIVAL	2019	URL	URL	R package + external solver	GPL‐3
NicheNet	2019	URL	URL	R package	GPL‐3
KPNN	2020	URL	URL	R and Python scripts	GPL‐3
CausalPath	2021	URL	URL	Java application	LGPL‐3

## A heterogeneous portfolio of methods, software, and applications

The 14 methods that fulfill these criteria are as follows: DEGraph (Jacob *et al*, 2012), CLIPPER (Martini *et al*, 2013), TEAK (Judeh *et al*, 2013), DEAP (Haynes *et al*, 2013), TieDIE (Paull *et al*, 2013), sub‐SPIA (Li *et al*, 2015), PHONEMES (Terfve *et al*, 2015), CausalR (Bradley & Barrett, 2017), HiPathia (Hidalgo *et al*, 2017), TPS (Köksal *et al*, 2018), CARNIVAL (Liu *et al*, 2019), NicheNet (Browaeys *et al*, 2020), KPNN (Fortelny & Bock, 2020), and CausalPath (Babur *et al*, 2021). We provide a brief description for each reviewed method in Box 3.

Box 3 **Methods' description**

In this box, we provide a brief description of all the methods considered in this Review. For more details, we refer interested readers to the original publication of each method. For the sake of homogeneity, we try to minimize all method‐specific terminology and use the term “statistical scores” to refer to scores obtained from statistical tests after contrasts or correlation analysis.

**DEGraph (Jacob *et al*,** **2012** **)**

DEGraph is a tool for the analysis of transcriptomics data and uses prior knowledge from KEGG or NCI in the form of pathways. It relies on a framework of multivariate test statistics for detecting differential expression patterns that are coherent with a given set of interactions. The pathway structure is leveraged to detect co‐regulated genes in high‐dimensional transcriptomics data and to subsequently reduce these to a lower‐dimensional space preserving most of the consistent distribution changes between two conditions and increasing statistical power. Hotelling's T^2^‐test is then applied to the low‐dimensional space to detect mean shifts between the multivariate normal distributions of two conditions. In a second step, DEGraph applies a branch‐and‐bound‐like algorithm for iterative testing and identification of significant subnetworks within the pathways.

**CLIPPER (Martini *et al*,** **2013** **)**

Based on its original framework TopologyGSA (Massa *et al*, 2010), CLIPPER finds biologically altered signaling pathways between two conditions using transcriptomics data. It obtains its prior knowledge via the graphite package, which retrieves pathways from KEGG, Panther, PathBank, PharmGKB, Reactome, SMPDB, and WikiPathways. CLIPPER uses Gaussian graphical models to compare the overall gene expression within a pathway as well as the interaction strengths between genes of a pathway. Both criteria are used to estimate the significance of the changes in pathways' functionality and are also applied to all subnetworks of the respective pathway to identify the relevant subgroups of genes in the network that drive these differences.

**TEAK (Judeh *et al*,** **2013** **)**

TEAK analyzes transcriptomics data and employs KEGG pathways as its main source of prior knowledge. It first applies a topological decomposition step to extract linear and non‐linear subnetworks from each pathway. Next, it applies Gaussian Bayesian networks to estimate conditional probability distributions at the subnetwork level. In these networks, each node is conceived as a linear combination of its parent nodes. TEAK differentiates two scenarios: Analysis of a single condition and contrasts. To rank subnetworks in the first scenario, it relies on the Bayesian Information Criterion (BIC), which is calculated from the maximum likelihood estimate of parameters. This score is calculated for each node and then summed to retrieve the final subnetwork score. In the contrast scenario, TEAK employs the Kullback–Leibler divergence of the two Bayesian networks, measuring the similarity of their joint probability distributions.

**DEAP (Haynes *et al*,** **2013** **)**

DEAP can be used to analyze transcriptomics data using pathways from KEGG and Reactome. It first maps statistical scores derived from differential expression analysis to PKN nodes. Next, DEAP iterates through the subnetworks of a pathway using an algorithm that prevents visiting the same node twice and which calculates a score for each subnetwork. This subnetwork score is based on the agreement between the sign of interactions and the sign of transcriptomic statistical scores. After evaluating all the subnetworks in a pathway, the maximum absolute subnetwork score for a given pathway is retained for statistical evaluation. To assess the significance of each pathway, a random rotation test is applied, a permutation‐like approach designed to increase statistical power in scenarios with complex experimental designs and low‐to‐moderate sample sizes.

**TieDIE (Paull *et al*,** **2013** **)**

TieDIE was released as a method to simultaneously analyze genomics and transcriptomics data, and it was employed later for the analysis of phosphoproteomics data as well (Drake *et al*, 2016). The PKN described in the original manuscript is derived from multiple resources, including the NCI‐PID and Reactome. TieDIE needs at least two sets of statistical scores derived from omics data that are mapped to nodes and which reflect the groups of molecules to be connected using the PKN. The core of TieDIE is composed by a heat diffusion approach that propagates the signal from a first set of nodes through the network, up to a certain path length. In particular, it uses the heat diffusion algorithm implemented in HotNet (Vandin *et al*, 2012). After obtaining the heat diffusion values for one of the sets, it runs the same process from the second set of nodes using a reversed PKN. Then, it retrieves a subnetwork that connects the two sets of input nodes using the diffusion scores from both processes. In a final step, it filters the subnetwork to retain sign‐consistent paths.

**sub‐SPIA** **(Li *et al*,** **2015** **)**

sub‐SPIA is an extension of the SPIA method (Tarca *et al*, 2009), which analyzes transcriptomics data using KEGG pathways. After a contrast analysis, differentially expressed genes are mapped to the network and subnetworks are extracted by the reconstruction of a minimal‐spanning tree, which retains the maximum number of closely connected differentially expressed nodes while minimizing the number of included non‐differentially expressed nodes. To subsequently identify the significantly altered subnetworks in the condition under study, the SPIA method combines the significance level of (i) an over‐representation analysis of the number of differentially expressed genes observed in the subnetwork with (ii) the significance level of a score that captures the topology of the pathways and the signature nodes by propagating measured (signed) expression changes across the pathway topology. This way, upstream nodes have more influence than downstream nodes and signals from consecutive nodes score higher than signals from individual unconnected nodes.

**PHONEMES (Terfve *et al*,** **2015** **)**

Originally released in 2015 (Terfve *et al*, 2015), and updated in 2021 (Gjerga *et al*, 2021), PHONEMES is a method to create models of signaling networks using phosphoproteomics data. It employs a directed PKN constructed from kinase‐substrate interactions from OmniPath (Türei *et al*, 2016). Additional nodes are also added in order to connect substrates (which are phosphosites) back to kinases. The processed input consists of a Boolean vector where the phosphoproteomic measurements are assigned a perturbed or control state and are then mapped to network nodes. In addition, users can also provide upstream perturbed nodes (e.g., drug targets). In the absence of such information, a kinase enrichment analysis is performed to determine kinase activities from phosphoproteomics data and use them as putative perturbed nodes. The current version of the method solves an optimization problem, which is conceived as a subnetwork extraction task. For this, PHONEMES translates the PKN to integer linear programming (ILP) constraints. To evaluate each possible subnetwork, PHONEMES employs an objective function that rewards the inclusion of perturbed nodes and penalizes the inclusion of control ones. This function also controls the final network size to prevent the obtention of gigantic solutions including all perturbed nodes.

**CausalR (Bradley & Barrett,** **2017** **)**

CausalR is a method to analyze transcriptomics data. This method was not released together with a PKN, but accepts any signed and directed PKN. It uses the sign of omics statistical scores as input. The core of CausalR is composed of two main steps: First, it calculates the number of correct, ambiguous, and incorrect shortest paths to omics statistical scores from all the nodes in the PKN, up to a predefined path length, by checking the sign consistency of the paths. Then, it repeats the same process for a varying number of path lengths and computes the significance of each master regulator subnetwork using the approach described in Chindelevitch et al. (Chindelevitch *et al*, 2012), which was designed to generate null distributions for causal graphs.

**HiPathia (Hidalgo *et al*,** **2017** **)**

HiPathia uses KEGG as its main source of prior knowledge to analyze transcriptomics data. Within the preprocessing steps, this tool divides each pathway into subnetworks that connect nodes with an in‐degree of zero to nodes with an out‐degree of zero, using an algorithm that relies on shortest paths. After this, HiPathia maps gene expression measurements to the nodes that form each subnetwork. Assuming an initial signal of 1 for each node with an in‐degree of zero, it applies an iterative signal propagation algorithm to calculate the signal that arrives at nodes with an out‐degree of zero. This algorithm calculates, for each node recursively, an activity value using the transcriptomic measurement of the node, if available, and the activity values of its parent nodes. In the case of loops, the iterative algorithm operates until the update in node activities is below a user‐defined threshold. The predicted signal matrix is then compared between samples of interest to rank the subnetworks and to generate mechanistic hypotheses.

**TPS (Köksal *et al*,** **2018** **)**

TPS allows the modeling of temporal relationships in signaling networks from phosphoproteomics time series data. The method uses three types of input: an undirected protein–protein interaction (PPI) network, phosphoproteomics time series data of a stimulus response and an optional PKN for interaction directions and signs. These inputs are used to construct a set of logical constraints that are expressed using linear integer arithmetics. The constraints are used to obtain all feasible signaling pathways that could explain the downstream signaling network from the stimulated source nodes using a symbolic solver. TPS starts from an undirected PPI network, which is first modified to a condition‐specific network by filtering out interactions that do not form critical connections between measured proteins, and including unobserved connective proteins from the background PPI network. It uses the source of stimulation and the highest magnitude fold change of a protein's phosphorylation peak as source of information. In a second step, data are discretized to a set of temporal constraints ensuring the correct order of signaling events. Third, the PKN is used for adding directionality and sign constraints to the edge topology of the PPI network. Subsequently, all valid individual networks are summarized into a single aggregate network, which is the graph union of all signed and directed networks satisfying the given set of constraints and representing the entire possible solution space.

**CARNIVAL (Liu *et al*,** **2019** **)**

CARNIVAL is a tool to analyze transcriptomics (Liu *et al*, 2019), but it can also be used for the analysis of multi‐omics data through COSMOS (Dugourd *et al*, 2021). CARNIVAL uses a PKN containing protein‐level signaling events derived from OmniPath. Its main inputs are statistical scores derived from transcriptomics data. First, transcription factor (TF) activities are inferred using DoRothEA (Garcia‐Alonso *et al*, 2019). Optionally, PROGENy (Schubert *et al*, 2018) can be used to calculate signaling pathway activities, which are mapped to PKN nodes. Additional information about nodes that are known to be perturbed can also be provided (e.g., drug targets). Similarly to PHONEMES, CARNIVAL first translates the PKN to integer linear programming constraints. Next, it solves an optimization problem whose objective function considers the mismatch between predicted and measured signaling activities (for TFs, PROGENy nodes and perturbations) and penalizes the total number of nodes in solutions This provides a subnetwork whose signal flow is as coherent as possible with the inferred signaling activities.

**NicheNet (Browaeys *et al*,** **2020** **)**

NicheNet is a method to predict the activity of ligands given a set of transcriptional targets. It also includes a functionality to retrieve the signaling subnetwork that connects a particular set of ligands with a set of downstream transcriptional regulators. Here, we focus on the latter. The PKN employed by NicheNet contains directed edges and was constructed through the integration of multiple resources. The resulting PKN is divided into two parts: A signaling network that connects ligands with transcriptional regulators (TFs) and a gene regulatory network (GRN) connecting such regulators with their targets. The core of NicheNet is a model that defines a regulatory potential of ligands over transcriptional targets. This model was optimized to tune parameters that: (i) control the contribution of each prior knowledge resource to edge weights, (ii) apply a hub correction factor in both parts of the PKN, and (iii) control the behavior of the algorithm that determines the closeness of ligands to transcriptional regulators (which is based on the PageRank algorithm (Page *et al*, 1999)). NicheNet requires users to provide a set of ligands and targets of interest. Given those, it first calculates a fixed number of ligand–regulator–target combinations that maximize the regulatory potential. Next, it retrieves all the genes involved in the shortest paths between selected ligands and inferred regulators. These shortest paths are computed maximizing again the regulatory potential and are obtained through the application of a modified version of Dijkstra's algorithm (Dijkstra, 1959). In a final step, NicheNet retrieves all the possible interactions between selected ligands, signaling mediators, regulators, and target genes from its PKN.

**KPNN (Fortelny & Bock,** **2020** **)**

KPNN can be employed to analyze (single‐cell) transcriptomics data. Similar to NicheNet, its PKN is divided into two parts: (i) Gene regulatory interactions connecting TFs with their targets and (ii) signaling interactions that connect receptors to TFs. This method uses prior knowledge to determine the topology of artificial neural networks, which are then trained on the prediction task of classifying a sample label of interest (e.g., experimental condition or cell type). While the networks reflect signal transmission from cellular receptors to transcriptional targets, they are used in the opposite direction during the training process: The status of an output node, which is connected to all receptors, is predicted using transcriptional targets as input. These input values are obtained from single‐cell transcriptomics measurements. KPNN adds certain tweaks to standard neural networks: (i) It performs dropouts of a proportion of input and hidden nodes during the training process and (ii) it trains the network using a control input that carries no signal. Node weights calculated from the training process with the real input (transcriptomics) are compared with the node weights obtained from control input, which account for certain PKN properties, such as the presence of hubs. The differential node weights are then used to interpret the importance of intermediate signaling nodes and to extract relevant subnetworks.

**CausalPath (Babur *et al*,** **2021** **)**

CausalPath retrieves causal interactions from phosphoproteomics data. It also accepts transcriptomics and proteomics statistical scores and can use them to evaluate interactions that capture gene regulatory events. It employs a PKN that was created using a graphical pattern search framework applied to diagrams from the Pathway Commons database (Rodchenkov *et al*, 2020). As processed data input, it employs a Boolean vector derived from the sign of the statistical scores. In its core, CausalPath employs logical equations to check the consistency between omics variables and the logic variables that describe each interaction in the PKN. Next, it keeps and retrieves those interactions consistent with the data and creates a subnetwork with all of them. The significance of extracted subnetworks is then examined through network permutation approaches that evaluate network size and the out‐degree of nodes.

As this field is relatively recent, the technical vocabulary and levels of mathematical detail diverge between publications. Similarly, the software packages accompanying the methods are different in form, ranging from software with graphical user interface to collections of R and Python scripts. There are multiple publications describing different versions of certain methods, as is the case for CLIPPER (Massa *et al*, 2010; Martini *et al*, 2013), HiPathia (Hidalgo *et al*, 2017; Rian *et al*, 2021) and PHONEMES (Terfve *et al*, 2015; Gjerga *et al*, 2021). Relatedly, there are also articles where the reviewed methods were expanded, refined, or applied to different data modalities, such as TieDIE (Paull *et al*, 2013) to phosphoproteomics (Drake *et al*, 2016), HiPathia (Hidalgo *et al*, 2017) to single‐cell transcriptomics (Falco *et al*, 2020), or CARNIVAL's core formulation (Liu *et al*, 2019) to multi‐omics in COSMOS (Dugourd *et al*, 2021). The underlying methods are very heterogeneous, and we decided to review them in terms of three major properties: (i) characteristics of input measurements, (ii) content of PKNs, and (iii) methodological properties, which we discuss in the following sections (see also Table 2.).

**Table 2:** Method properties. The table represents a binary matrix. Each method (columns) can have (X) or not (‐) a determined property (rows).

Omics data properties		DEGraph	CLIPPER	TEAK	DEAP	TieDIE	sub‐SPIA	PHONEMES	CausalR	HiPathia	TPS	CARNIVAL	NicheNet	KPNN	CausalPath
Omics layers	Transcriptomics	X	X	X	X	X	X	‐	X	X	‐	X	X	X	X
	Phosphoproteomics	‐	‐	‐	‐	X	‐	X	‐	‐	X	‐	‐	‐	X
Biological resolution	Bulk	X	X	X	X	X	X	X	X	X	X	X	X	‐	X
	Single‐cell	‐	‐	‐	‐	‐	‐	‐	‐	X	‐	‐	X	X	‐
Time resolution	Time series data	‐	‐	‐	‐	‐	‐	X	‐	‐	X	‐	‐	‐	‐
Omics data used as	Measurements (observations)	‐	‐	X	‐	‐	‐	‐	‐	X	X	‐	‐	X	‐
	Statistical scores (contrast/correlation)	X	X	‐	X	X	X	X	X	‐	X	X	X	‐	X
Numerical input	Continuous	X	X	X	X	X	X	‐	‐	X	X	X	‐	X	‐
	Discrete	‐	‐	‐	‐	‐	‐	‐	X	‐	‐	‐	‐	‐	‐
	Boolean	‐	‐	‐	‐	‐	‐	X	‐	‐	X	‐	X	‐	X
Additional input	Does not use additional inputs	X	X	X	X	‐	X	‐	X	X	‐	‐	‐	X	‐
	Accepts additional inputs	‐	‐	‐	‐	‐	‐	X	‐	‐	‐	X	X	‐	X
	Requires additional inputs	‐	‐	‐	‐	X	‐	‐	‐	‐	X	‐	‐	‐	‐
PKN properties															
Sign	Directed	‐	X	X	‐	‐	X	X	‐	‐	X	‐	X	X	‐
	Activations/Inhibitions	X	‐	‐	X	X	‐	‐	X	X	X	X	‐	‐	X
Size	Pathways	X	X	X	X	‐	X	‐	‐	X	‐	‐	‐	‐	‐
	Large networks	‐	‐	‐	‐	X	‐	X	X	‐	X	X	X	X	X
Biological content	Protein signaling interactions	X	X	X	X	X	X	X	‐	X	X	X	X	X	X
	Gene regulatory interactions	X	X	X	X	‐	X	‐	‐	X	‐	‐	X	X	X
Method properties															
Omics to PKN	Direct mapping to nodes	X	X	X	X	‐	X	X	X	X	X	‐	X	X	X
	Indirect mapping to nodes	‐	‐	‐	‐	X	‐	‐	‐	‐	‐	X	‐	‐	‐
Not‐measured nodes	Estimates unmeasured nodes state	X	‐	‐	‐	X	‐	X	X	X	X	X	‐	X	‐
	Includes unmeasured nodes in output	X	X	X	‐	X	X	X	X	X	X	X	X	X	‐
Loops	Models feedforward loops	‐	‐	X	‐	‐	‐	‐	‐	X	X	‐	‐	‐	‐
	Models feedback loops	‐	‐	‐	‐	‐	‐	X	‐	X	‐	‐	‐	‐	‐
	Detects and removes feedforward loops	‐	X	‐	‐	‐	‐	‐	‐	‐	‐	‐	‐	‐	‐
	Detects and removes feedback loops	‐	X	X	X	‐	‐	‐	‐	‐	‐	X	‐	X	‐
	Includes feedforward loops in output	X	X	X	‐	X	X	X	‐	X	X	X	X	X	X
	Includes feedback loops in output	‐	‐	‐	‐	X	X	X	‐	X	X	‐	X	‐	X
Algorithm type	Edge filtering and shortest path	‐	‐	‐	X	‐	‐	‐	X	‐	‐	‐	X	‐	X
	Recursive signal propagation and heat diffusion	‐	‐	‐	‐	X	‐	‐	‐	X	‐	‐	‐	‐	‐
	Graph theory and statistical testing	X	X	‐	‐	‐	X	‐	‐	‐	‐	‐	‐	‐	‐
	Bayesian networks	‐	‐	X	‐	‐	‐	‐	‐	‐	‐	‐	‐	‐	‐
	Integer linear programming	‐	‐	‐	‐	‐	‐	X	‐	‐	X	X	‐	‐	‐
	Neural networks	‐	‐	‐	‐	‐	‐	‐	‐	‐	‐	‐	‐	X	‐

## Molecular readouts: Transcriptomics and phosphoproteomics

Omics technologies provide a molecular snapshot of the biological system under study. In the context of this Review, both transcriptomics and phosphoproteomics measurements are used to estimate the activity levels of proteins. Four methods, PHONEMES, TieDIE, CausalPath, and TPS, were designed for or applied to phosphoproteomics, while the remaining ten methods were designed for the analysis of transcriptomics data. In addition, TieDIE, CausalPath, and CARNIVAL (COSMOS) were also applied to simultaneously analyze transcriptomics and phosphoproteomics data.

The number of available methods for the analysis of phosphoproteomics data, which captures signaling more directly, is smaller than for transcriptomics. This may be explained by data availability: Transcriptomics has offered genome‐wide coverage for more than 20 years, first with microarrays and later with RNA sequencing (RNA‐Seq) (Lowe *et al*, 2017), while reliable phosphoproteome‐based measurements at scale are a more recent development. Most untargeted and large‐scale measurements of the phosphoproteome are currently obtained through MS‐based approaches (Aebersold & Mann, 2016), quantifying the abundance of peptides that carry one or multiple phosphorylations. On human samples, modern protocols offer coverage above 5,000 phosphoproteins (proteins where at least one phosphorylation is detected) with rapidly decreasing costs and sample requirements (Bekker‐Jensen *et al*, 2020; Satpathy *et al*, 2020), but are overall still behind transcriptomic techniques in terms of coverage (10,000–20,000 messenger RNAs, depending on sequencing depth).

Some methods can exploit different levels of resolution in the input omics data. Most approaches were applied to omics data generated from bulk samples, which represent a compound signal that summarizes the status of multiple cells or cell types. In contrast, HiPathia, NicheNet, and KPNN were applied to data obtained at single‐cell resolution. Both TPS and PHONEMES contain algorithms designed to deal with time series observations. These exploit the temporal order of measurements to create hypotheses based on the dynamic nature of cell signaling, a fundamental property of cellular regulation (see Box 1). Of note, although TPS and PHONEMES employ an *ad hoc* strategy to deal with time‐resolved data, any method that performs contrast tests can potentially be applied to time series data if these are used as sets of pairwise comparisons, as shown in CausalPath.

The methods use data which, after initial preprocessing, define one value for each possible combination of sample (observation) and gene or phosphosite (feature) (e.g., normalized gene expression matrices derived from RNA sequencing). While TEAK, HiPathia, and KPNN can be directly applied to such data (referred to as “measurements”), all other methods require additional preprocessing, typically in the form of statistical tests using contrasts or correlations. The results of these statistical tests are then used as inputs (referred to as “scores” from here on). TPS employs both omics measurements and statistical scores. The additional preprocessing may introduce biases through the selection of the tests to be carried out or through the choice of thresholds (Wicherts *et al*, 2016). However, methods that do not require this step may also add biases in the post‐modeling analysis, as these methods also require users to select certain thresholds, such as the number of top‐ranked relevant networks. Most of the approaches that take measurements as input cannot work with statistical scores, creating a methodological barrier that makes it difficult to compare the methods.

Regardless of the level of preprocessing, the input data can be employed in a continuous scale or discretized. This is usually related to the numeric form that each method uses to model signaling, although it does not always coincide (e.g., CARNIVAL models node signaling states as discrete values, but transcriptomics scores are employed in a continuous form in its objective function). Some discrete measurements take the form of the sign of the contrast/correlation scores, namely {−1, 0, 1} for down‐regulation (or negative correlation), no change (no correlation), and up‐regulation (positive correlation), respectively. Another common form is to simply assign either a True or a False state (i.e., Boolean variables), which indicates whether a gene or phosphosite is considered perturbed or not. Data discretization connects with the abstraction process described above and in Box 1, and is a common strategy to scale the models to the large amount of molecular data generated by omics techniques, at the price of losing information.

Finally, certain methods can use additional inputs apart from omics data. We do not refer here to external information regarding observations (e.g., samples' experimental group), but rather to information that can be used to reduce the complexity of the modeling problem. For instance, NicheNet asks users for a set of ligands and transcriptional targets that are then connected through its PKN using shortest paths. The set of interesting ligands can be either selected using NicheNet's framework to predict ligand activities, or directly provided by the user. CARNIVAL and PHONEMES can use information about proteins that are known to be perturbed and that initiate the signal transmission, for example, drug targets or genetic perturbations. Finally, TieDIE and TPS require additional input data that represent the source of perturbation to be connected with measured nodes through the PKN. In TieDIE, these additional inputs can be obtained from other omics layers from the same or related samples (e.g., somatic mutations from genomics). All other methods only use measurements and scores derived from transcriptomics or phosphoproteomics data.

## The sources of prior knowledge

PKNs summarize many, typically thousands, known biochemical interactions extracted from previous experiments, which can have different levels of supporting evidence and curation. For example, the physical interactions retrieved from BioGRID can be supported by up to 17 different types of experimental evidence, including categories like “co‐localization” or “biochemical activity.” In contrast, there are databases that contain interactions generated from predictive approaches, which are rarely backed by confirmatory experimental evidence. For instance, NetworKIN predicts kinase–substrate interactions based on sequence similarity searches and protein–protein interactions (Linding *et al*, 2008). Most of the interactions that compose the PKNs employed by the reviewed methods fall in the first category (databases with curated interactions), although some methods such as TPS and CausalR do not mention specific sources of prior knowledge.

The PKNs used by the methods vary in both size and content. This is partly because they are derived from different databases, but also because they are preprocessed differently by each method. First, DEGraph, DEAP, TieDIE, CausalR, HiPathia, CARNIVAL, and CausalPath employ signed PKNs, where interactions are labeled either as “activations” or “inhibitions” (Fig 2). In contrast, CLIPPER, TEAK, sub‐SPIA, NicheNet, KPNN, and PHONEMES use unsigned but directed PKNs. TPS is a special case, as it can be run without prior knowledge about the direction or sign of interactions as well as with a partially or completely directed and/or signed network. The sign of interactions provides additional mechanistic insights about signaling events, but also increases the complexity of the modeling process (see Box 1). This prevents its direct incorporation in some of the methods. TieDIE and TPS tackle this using a hybrid approach: the sign of interactions is considered in a second step after a process of subnetwork extraction that ignores it.

Regarding the sources of prior knowledge networks, there are two categories: (i) methods that use pathway‐derived networks and (ii) methods that use large input networks. DEGraph, CLIPPER, TEAK, DEAP, sub‐SPIA, and HiPathia use networks from pathway databases such as KEGG or WikiPathways. The remaining methods use large networks retrieved from databases such as BioGRID, or SIGNOR, or meta‐resources such as OmniPath (Türei *et al*, 2016). The networks encoded by pathways are usually smaller than those that can be retrieved from interactome‐like resources. However, when the pathways are put together, they can form networks of similar size. This classification impacts the choice and limitations of computational approaches and also creates two categories of methods, which are rarely benchmarked together.

Finally, the biological content that PKNs provide can be classified into two major groups: (i) PKNs containing only protein signaling interactions and (ii) PKNs containing protein signaling interactions and gene regulatory interactions. Networks derived from pathway databases usually combine the two types of interactions, and hence methods using them that do not exclude gene regulatory interactions consider both types. Moreover, NicheNet, CausalPath, and KPNN also distinguish between more detailed categories, such as ligand–receptor interactions and enzyme–substrate interactions. NicheNet and KPNN use this information to establish a hierarchy within their PKNs: The signal is theoretically initiated by ligands and propagated through receptors and intermediate proteins to transcription factors (TFs). Then, the TF layer transmits the information to downstream targets through gene regulatory interactions. This matches the hierarchical way signaling is commonly conceived, but also simplifies a process that is known to be non‐linear and strongly affected by feedback mechanisms (see Box 1).

## Method properties: Input processing and approaches to model signaling

A common first step across methods is the mapping of omics measurements to nodes in the PKN. DEGraph, CLIPPER, TEAK, DEAP, sub‐SPIA, CausalR, HiPathia, TPS, NicheNet, KPNN, CausalPath, and PHONEMES use a direct mapping strategy and match each molecular feature to a node. Of those, KPNN, NicheNet, and CausalPath map transcriptomics data to nodes that represent transcriptional targets (downstream of TFs); and DEGraph, CLIPPER, TEAK, DEAP, sub‐SPIA, CausalR, and HiPathia directly map measurements and scores to protein nodes. By doing so, they assume that gene expression measurements are good proxies of the signaling activity of proteins. This is often not the case, given the number of intermediate, highly dynamic steps that occur between gene regulation and protein activation (Liu *et al*, 2016; Buccitelli & Selbach, 2020; Szalai & Saez‐Rodriguez, 2020). For this reason, TieDIE and CARNIVAL use a strategy based on indirect mapping and employ molecular measurements to predict the activity levels of upstream proteins, such as transcription factors or kinases (Dugourd & Saez‐Rodriguez, 2019). These activities are then mapped to PKN nodes. This way, these methods assume that the signaling activity of upstream proteins can be estimated from the observed molecular profiles.

Omics techniques may not provide genome‐wide coverage. At the network level, this often results in nodes for which no measurements are available. All reviewed methods except DEAP and CausalPath tolerate unmeasured nodes. However, not all the methods actively estimate signaling activities of such nodes and simply include them as a result of network aggregation/selection processes. This is the case for CLIPPER, TEAK, sub‐SPIA, and NicheNet.

A key feature of signaling networks is the presence of feedback and feedforward loops that can play major regulatory roles. While not all methods are able to deal with them, some may include them in their output. Specifically, we define three categories of methods regarding loops: (i) able to model loops, (ii) able to detect and remove loops, and (iii) able to include loops in their output networks. All methods except DEAP and CausalR can include feedforward loops in their output, although only TEAK, HiPathia, and TPS provide a framework to actively model them. Similarly, all methods except TEAK, DEAP, CausalR, CARNIVAL, and KPNN can output networks with feedback loops, while only PHONEMES and HiPathia actively model them.

**Figure 3:**
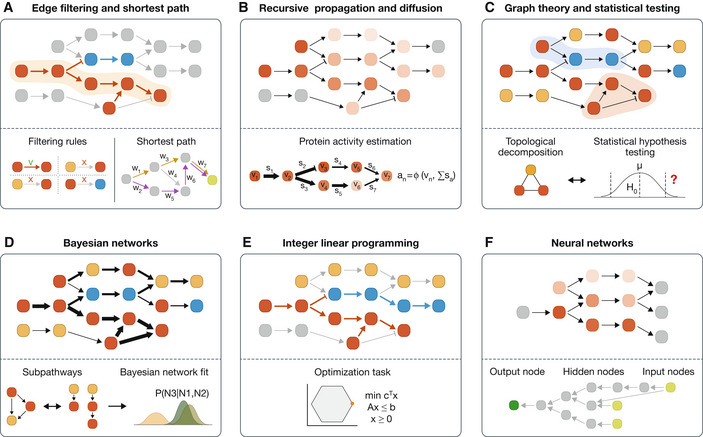
Six categories of computational approaches to model cellular signaling (A) Edge filtering and shortest path, (B) recursive signal propagation and heat diffusion, (C) graph theory and statistical testing, (D) Bayesian networks, (E) integer linear programming, and (F) neural networks. The upper part of each panel shows the solution of a toy network obtained by each formalism, whereas the bottom panel illustrates its generalized method. Nodes depict proteins, arrows activating and bars inhibiting interactions. Upper panels: Yellow indicates a protein with an unchanged activity state in comparison with a control condition, red an activated, and blue a repressed state. All proteins and interactions that are not part of the solution network are depicted in gray. (A) Only consistent interactions are kept according to the filtering rules. The activities of interactions are inferred from the PKN and the measured activity states of the connected proteins. The shortest signaling path (upper panel, yellow cloud) between an initial protein and a target protein can be calculated based on distance (brown) or a combination of weights (w_i_) and distance (purple) (bottom panel). (B) A protein’s activity estimate a_n_ in the solution network (upper panel) is calculated as a function f() of its own measured value v_i_ (e.g., based on transcriptomics data) and the sum of all incoming activity signals s_i_ (bottom panel) that it receives from upstream proteins according to the PKN. (C) After topological decomposition, all identified subnetworks of the PKN are tested for an enrichment of measured protein activity states taking the subnetwork topology into account. In the upper panel, the blue and red clouds indicate subnetworks that have underrepresented and overrepresented protein activities, respectively. (D) Linear subnetworks are extracted from a PKN and subjected to a Bayesian network fitting measured protein activity states. Arrow thickness in the solution network corresponds to the assigned probability of the interaction activity. (E) Integer linear programming outputs the optimal signaling subnetwork consistent with the PKN between an initial protein and a target protein by minimizing the discrepancy between protein activity states and interaction constraints given by the PKN. (F) Knowledge‐primed neural networks model the importance of proteins between receptors and transcriptional targets (hidden nodes, bottom panel). Edge direction in the bottom panel is inverted to represent that the training occurs from transcriptional targets (input nodes, right bottom panel) to receptors (output nodes, left bottom panel).

Finally, the strategies employed to model signal transmission are heterogeneous, ranging from shortest paths to complex neural networks. We divided the approaches into six categories (see Fig 3):

1 Edge filtering and shortest path (Fig 3A). This category includes CausalR, DEAP, NicheNet, and CausalPath. CausalPath and DEAP iterate through PKN interactions and select those that are consistent with omics scores. On the contrary, CausalR and NicheNet connect upstream regulators to downstream targets using algorithms that calculate shortest paths.
2 Recursive signal propagation and heat diffusion (Fig 3B), including TieDIE and HiPathia. Both approaches initialize the signal in a given set of nodes and then simulate its propagation through the network. On each iteration, a method‐specific function determines how the signal is propagated to adjacent nodes.
3 Graph theory and statistical testing (Fig 3C), including DEGraph, CLIPPER, and sub‐SPIA. All the methods in this category are based on graph theory approaches, optionally in combination with statistical procedures.
4 Bayesian networks (Fig 3D), including only TEAK. Bayesian networks can be applied to model signaling through conditional dependencies. In TEAK, each node is conceived as a linear combination of its parent nodes in a directed acyclic graph.
5 Integer linear programming (Fig 3E), including TPS, CARNIVAL, and PHONEMES. Methods in this category translate PKNs and molecular measurements into integer linear programming constraints that allow subnetwork extraction tasks to be carried out.
6 Neural networks (Fig 3F), including only KPNN. This method tunes the topology of a neural network to represent prior knowledge. It then trains the neural network under supervised learning settings, computes node importance, and uses these values to interpret the model and extract relevant subnetworks.

Certain methods, such as TieDIE or TPS, consist of multiple steps. For the classification described above, we focused on the specific part of the method that models signal propagation. TPS employs either a *satisfiability modulo theories* solver or a custom solver. However, we included it in the “integer linear programming” category because of its similarity to these approaches. More information about each of the methods can be found in Table 2. and Box 3.

## Challenges and outlook

In this Review, we explore methods able to generate hypotheses about signaling mechanisms in the form of networks. Traditionally, this was done with experiments of limited molecular coverage that were interpreted via manual bibliographic curation. Omics technologies have changed this paradigm by measuring thousands of molecules in a single assay. While this presents an opportunity to systematically leverage prior knowledge to analyze omics data, many questions remain open.

### Benchmarking

Benchmarking studies are essential to understand the advantages and limitations of novel computational approaches for the analysis of omics data (Weber *et al*, 2019). Most benchmarking strategies define metrics based on ground truth data that are used to score novel methods. In machine learning, it is a common practice to test new approaches on a large and diverse set of publicly available datasets from open repositories such as OpenML (Vanschoren *et al*, 2014). However, this practice is less common in network modeling, and the ground truth is usually generated from prior knowledge, simulated data, or perturbation experiments. When ground truth is established using prior knowledge, benchmark studies make the assumption that our understanding of a biological system is sufficient to determine which nodes and edges are true positives. This assumption may not hold in many scenarios, and, as an alternative, simulated data or perturbation experiments are used. Large efforts have been made to generate perturbation data in consortia such as the Library of Integrated Network‐Based Cellular Signatures (Koleti *et al*, 2018). However, generating perturbation data is costly and not possible for all contexts and tasks (e.g., when working with patient samples (Saez‐Rodriguez & Blüthgen, 2020)). In contrast, while simulated data can provide ground truth in most scenarios, it is only an approximation of the biological reality and is inherently biased and limited by our understanding of the underlying molecular processes.

Most independent benchmarks (not carried out by method developers) were restricted to certain types of methods. For instance, (Ihnatova *et al*, 2018) evaluated several methods that consider the topological properties of pathways, including CLIPPER, DEGraph, and SPIA (Tarca *et al*, 2009). Another study benchmarked methods that extract causal signaling subnetworks from transcriptomics data in response to drug perturbation (Hosseini‐Gerami *et al*, 2022). CausalR and CARNIVAL were among the methods evaluated in this benchmark. We could not find an independent study that benchmarked the methods described in this Review. Overall, the field would benefit from more benchmarks and, given the challenges to obtain ground truth data, we believe that an extensive catalog of simulated data would enable further developments.

### The limitations of prior knowledge networks

Despite major progress, our knowledge of cell signaling is still limited. This is exemplified in the field of post‐translational protein modifications: phosphorylation events are known to play a fundamental role in cellular signaling, but we only know the function (e.g., activatory or inhibitory) of less than 5% of the known phosphorylations that occur in human cells (Needham *et al*, 2019; Ochoa *et al*, 2020). Furthermore, more than 90% of those known phosphorylation events occur in a subset of particularly well‐studied kinases (20%). At the network level, a small proportion of well‐studied proteins accounts for the majority of interactions (see Box 2). It is still unclear whether this is an inherent property of biological networks, or whether it is a product of annotation preferences introduced by humans (Haynes *et al*, 2018). Heavily studied genes have been associated with a higher amount of biological functions, and this multifunctionality may introduce a bias when interpreting the outcome of computational models based on omics measurements and PKNs (Gillis & Pavlidis, 2011; Weidemüller *et al*, 2021; Kustatscher *et al*, 2022). Of the methods included in this Review, TieDIE, NicheNet, and KPNN explicitly account for the presence of hubs in PKNs. TieDIE's heat diffusion algorithm compensates input and output heat on hubs, while NicheNet and KPNN employ a hub correction factor and a control run on data carrying no signal, respectively.

Another major limitation of prior knowledge is that it is not directly transferable between different biological contexts, despite it being common practice. Experimental evidence supporting PKN interactions is usually obtained under very specific conditions (e.g., in a tissue or cell line), and there is no guarantee that these interactions also occur outside these conditions. Furthermore, most of our knowledge about signaling has been generated in a narrow set of biological contexts (see Box 2).

On a final note, we do not yet have an accurate estimate of how much information we are missing in our current prior knowledge. We may be trying to model signaling by taking into account only a small fraction of the interactions that actually occur between proteins and other molecules. In general, when trying to understand signaling, we must consider all the limitations of prior knowledge, as they may provide a partial explanation for the caveats of our models.

### Higher resolution to better understand cellular signaling

New developments in omics technologies allow us to look at certain biological processes at new levels of resolution and coverage. Since their inception (Tang *et al*, 2009), single‐cell sequencing technologies have evolved rapidly to provide reasonable (if not deep) transcriptome coverage for large numbers of individual cells (Picelli *et al*, 2014). This technology has been used to uncover some of the biological heterogeneity that cannot be studied using omics data from bulk samples. Furthermore, it has drastically increased the availability of observations, enabling the application of methods such as neural networks (Fortelny & Bock, 2020; preprint: Nilsson *et al*, 2021). Relatedly, novel single‐cell untargeted proteomics protocols are rapidly evolving (Perkel, 2021). Although coverage is lower than in single‐cell transcriptomics, if single‐cell proteomics reaches a sufficient degree of maturity to comprehensively measure phosphopeptides, it will provide invaluable data to model cell signaling capturing cellular heterogeneity.

In addition to single‐cell technologies, novel untargeted proteomic approaches like limited proteolysis proteomics (Feng *et al*, 2014) and thermal proteome profiling proteomics (Savitski *et al*, 2014) allow us to estimate different types of regulatory events by measuring protein conformational changes. Both approaches were recently applied to study signaling cascades, demonstrating their ability to capture protein‐driven signaling (Potel *et al*, 2021; Cappelletti *et al*, 2021). Although exploiting proteomic conformational readouts to predict signaling functions is not a simple task, we believe that these technologies will complement existing technologies. In conclusion, advances in measurement techniques offer new perspectives to study signaling at scale, and computational methods must keep pace to enable the extraction of mechanistic insights from new data.

### Deciphering signaling networks to improve personalized treatments

All reviewed methods aim to generate testable hypotheses to ultimately improve our understanding of complex biological systems. In the context of disease, this could translate to better treatment selection and prioritization for pharmacological interventions that target signaling components (Saez‐Rodriguez & Blüthgen, 2020). However, for the integration into clinical practice, such methods must demonstrate that they can outperform the techniques currently used to select treatments. At the same time, this also requires an infrastructure in which, for each patient, omics data and subsequent models can be generated in a cost‐ and time‐effective manner. If the costs of generating omics data continue to fall, and the computational efficiency of the methods continues to increase, we expect that methods that model cellular signaling will occupy a central role in the translation of omics data into personalized treatment choices.

## Conclusions

The methods we reviewed aim to extract mechanistic insights about signaling through the integration of high‐coverage molecular measurements and prior knowledge. Their output is a network (or a ranking of networks) that may be relevant in the specific biological context captured through omics measurements. This work complements recent reviews of approaches that model other types of biological networks, such as metabolic reaction networks (Cruz *et al*, 2020; Hrovatin *et al*, 2022) and gene regulatory networks (Barbuti *et al*, 2020). We reviewed 14 recent methods that we found to be very diverse and classified them according to different characteristics, including omics data, prior knowledge, and methodological properties. As one of the main challenges yet to overcome, we believe that the field lacks benchmark studies to determine the validity of the many assumptions made by each method. In addition, we encourage users to maintain a conservative attitude toward model results, since the biases of prior knowledge, together with the limitations of omics technologies and model assumptions, can make predictions that differ greatly from biological reality. In terms of outlook, we expect these methods to gain relevance thanks to new omics technologies, such as single‐cell proteomics and techniques able to measure protein conformational changes, and we believe that they will become key tools in the precision medicine of the future.

## Author contributions

**Martin Garrido‐Rodriguez:** Conceptualization; investigation; visualization; writing – original draft; writing – review and editing. **Katharina Zirngibl:** Investigation; visualization; writing – original draft; writing – review and editing. **Olga Ivanova:** Writing – original draft. **Sebastian Lobentanzer:** Writing – original draft; writing – review and editing. **Julio Saez‐Rodriguez:** Conceptualization; supervision; funding acquisition; writing – original draft; writing – review and editing.

## Disclosure and competing interests statement

JSR reports funding from GSK and Sanofi and fees from Travere Therapeutics and Astex.

References

Abou‐Jaoudé
W
, 
Traynard
P
, 
Monteiro
PT
, 
Saez‐Rodriguez
J
, 
Helikar
T
, 
Thieffry
D
, 
Chaouiya
C
 (2016) Logical modeling and dynamical analysis of cellular networks. Front Genet
7: 94
2730343410.3389/fgene.2016.00094PMC4885885

Aebersold
R
, 
Mann
M
 (2016) Mass‐spectrometric exploration of proteome structure and function. Nature
537: 347–355
2762964110.1038/nature19949

Aoki
K
, 
Yoshida
K
 (2017) Biological consequences of priming phosphorylation in cancer development. In Protein Phosphorylation, 

C
Prigent

 (ed). London: InTech


Babur
Ö
, 
Luna
A
, 
Korkut
A
, 
Durupinar
F
, 
Siper
MC
, 
Dogrusoz
U
, 
Vaca Jacome
AS
, 
Peckner
R
, 
Christianson
KE
, 
Jaffe
JD

*et al* (2021) Causal interactions from proteomic profiles: molecular data meet pathway knowledge. Patterns (N Y)
2: 100257
3417984310.1016/j.patter.2021.100257PMC8212145

Barbuti
R
, 
Gori
R
, 
Milazzo
P
, 
Nasti
L
 (2020) A survey of gene regulatory networks modelling methods: from differential equations, to Boolean and qualitative bioinspired models. J Membr Comput
2: 207–226


Bekker‐Jensen
DB
, 
Bernhardt
OM
, 
Hogrebe
A
, 
Martinez‐Val
A
, 
Verbeke
L
, 
Gandhi
T
, 
Kelstrup
CD
, 
Reiter
L
, 
Olsen
JV
 (2020) Rapid and site‐specific deep phosphoproteome profiling by data‐independent acquisition without the need for spectral libraries. Nat Commun
11: 787
3203416110.1038/s41467-020-14609-1PMC7005859

Blazek
M
, 
Santisteban
TS
, 
Zengerle
R
, 
Meier
M
 (2015) Analysis of fast protein phosphorylation kinetics in single cells on a microfluidic chip. Lab Chip
15: 726–734
2542871710.1039/c4lc00797b

Bradley
G
, 
Barrett
SJ
 (2017) CausalR: extracting mechanistic sense from genome scale data. Bioinformatics
33: 3670–3672
2866636910.1093/bioinformatics/btx425PMC5870775

Brandman
O
, 
Meyer
T
 (2008) Feedback loops shape cellular signals in space and time. Science
322: 390–395
1892738310.1126/science.1160617PMC2680159

Browaeys
R
, 
Saelens
W
, 
Saeys
Y
 (2020) NicheNet: modeling intercellular communication by linking ligands to target genes. Nat Methods
17: 159–162
3181926410.1038/s41592-019-0667-5

Buccitelli
C
, 
Selbach
M
 (2020) mRNAs, proteins and the emerging principles of gene expression control. Nat Rev Genet
21: 630–644
3270998510.1038/s41576-020-0258-4
Cancer Genome Atlas Research Network
, 
Weinstein
JN
, 
Collisson
EA
, 
Mills
GB
, 
Shaw
KRM
, 
Ozenberger
BA
, 
Ellrott
K
, 
Shmulevich
I
, 
Sander
C
, 
Stuart
JM
 (2013) The cancer genome atlas pan‐cancer analysis project. Nat Genet
45: 1113–1120
2407184910.1038/ng.2764PMC3919969

Cappelletti
V
, 
Hauser
T
, 
Piazza
I
, 
Pepelnjak
M
, 
Malinovska
L
, 
Fuhrer
T
, 
Li
Y
, 
Dörig
C
, 
Boersema
P
, 
Gillet
L

*et al* (2021) Dynamic 3D proteomes reveal protein functional alterations at high resolution in situ. Cell
184: 545–559
3335744610.1016/j.cell.2020.12.021PMC7836100

Chindelevitch
L
, 
Loh
P‐R
, 
Enayetallah
A
, 
Berger
B
, 
Ziemek
D
 (2012) Assessing statistical significance in causal graphs. BMC Bioinformatics
13: 35
2234844410.1186/1471-2105-13-35PMC3307026

Cruz
F
, 
Faria
JP
, 
Rocha
M
, 
Rocha
I
, 
Dias
O
 (2020) A review of methods for the reconstruction and analysis of integrated genome‐scale models of metabolism and regulation. Biochem Soc Trans
48: 1889–1903
3294065910.1042/BST20190840

Deribe
YL
, 
Pawson
T
, 
Dikic
I
 (2010) Post‐translational modifications in signal integration. Nat Struct Mol Biol
17: 666–672
2049556310.1038/nsmb.1842

Dijkstra
EW
 (1959) A note on two problems in connexion with graphs. Numer Math
1: 269–271


Drake
JM
, 
Paull
EO
, 
Graham
NA
, 
Lee
JK
, 
Smith
BA
, 
Titz
B
, 
Stoyanova
T
, 
Faltermeier
CM
, 
Uzunangelov
V
, 
Carlin
DE

*et al* (2016) Phosphoproteome Integration reveals patient‐specific networks in prostate cancer. Cell
166: 1041–1054
2749902010.1016/j.cell.2016.07.007PMC4985183

Dugourd
A
, 
Kuppe
C
, 
Sciacovelli
M
, 
Gjerga
E
, 
Gabor
A
, 
Emdal
KB
, 
Vieira
V
, 
Bekker‐Jensen
DB
, 
Kranz
J
, 
EMJ
B

*et al* (2021) Causal integration of multi‐omics data with prior knowledge to generate mechanistic hypotheses. Mol Syst Biol
17: e9730
3350208610.15252/msb.20209730PMC7838823

Dugourd
A
, 
Saez‐Rodriguez
J
 (2019) Footprint‐based functional analysis of multiomic data. Curr Opin Syst Biol
15: 82–90
3268577010.1016/j.coisb.2019.04.002PMC7357600

Edwards
AM
, 
Isserlin
R
, 
Bader
GD
, 
Frye
SV
, 
Willson
TM
, 
Yu
FH
 (2011) Too many roads not taken. Nature
470: 163–165
2130791310.1038/470163a

Ellis
MJ
, 
Gillette
M
, 
Carr
SA
, 
Paulovich
AG
, 
Smith
RD
, 
Rodland
KK
, 
Townsend
RR
, 
Kinsinger
C
, 
Mesri
M
, 
Rodriguez
H

*et al* (2013) Connecting genomic alterations to cancer biology with proteomics: the NCI Clinical Proteomic Tumor Analysis Consortium. Cancer Discov
3: 1108–1112
2412423210.1158/2159-8290.CD-13-0219PMC3800055

Falco
MM
, 
Peña‐Chilet
M
, 
Loucera
C
, 
Hidalgo
MR
, 
Dopazo
J
 (2020) Mechanistic models of signaling pathways deconvolute the glioblastoma single‐cell functional landscape. NAR Cancer
2: zcaa011
3431668610.1093/narcan/zcaa011PMC8210212

Feng
Y
, 
De Franceschi
G
, 
Kahraman
A
, 
Soste
M
, 
Melnik
A
, 
Boersema
PJ
, 
de Laureto
PP
, 
Nikolaev
Y
, 
Oliveira
AP
, 
Picotti
P
 (2014) Global analysis of protein structural changes in complex proteomes. Nat Biotechnol
32: 1036–1044
2521851910.1038/nbt.2999

Fortelny
N
, 
Bock
C
 (2020) Knowledge‐primed neural networks enable biologically interpretable deep learning on single‐cell sequencing data. Genome Biol
21: 190
3274693210.1186/s13059-020-02100-5PMC7397672

Garcia‐Alonso
L
, 
Holland
CH
, 
Ibrahim
MM
, 
Turei
D
, 
Saez‐Rodriguez
J
 (2019) Benchmark and integration of resources for the estimation of human transcription factor activities. Genome Res
29: 1363–1375
3134098510.1101/gr.240663.118PMC6673718

Gerosa
L
, 
Chidley
C
, 
Fröhlich
F
, 
Sanchez
G
, 
Lim
SK
, 
Muhlich
J
, 
Chen
J‐Y
, 
Vallabhaneni
S
, 
Baker
GJ
, 
Schapiro
D

*et al* (2020) Receptor‐driven ERK pulses reconfigure MAPK signaling and enable persistence of drug‐adapted BRAF‐Mutant melanoma cells. Cell Syst
11: 478–494
3311335510.1016/j.cels.2020.10.002PMC8009031

Gillespie
M
, 
Jassal
B
, 
Stephan
R
, 
Milacic
M
, 
Rothfels
K
, 
Senff‐Ribeiro
A
, 
Griss
J
, 
Sevilla
C
, 
Matthews
L
, 
Gong
C

*et al* (2022) The reactome pathway knowledgebase 2022. Nucleic Acids Res
50: D687–D692
3478884310.1093/nar/gkab1028PMC8689983

Gillis
J
, 
Pavlidis
P
 (2011) The impact of multifunctional genes on “guilt by association” analysis. PLoS One
6: e17258
2136475610.1371/journal.pone.0017258PMC3041792

Gjerga
E
, 
Dugourd
A
, 
Tobalina
L
, 
Sousa
A
, 
Saez‐Rodriguez
J
 (2021) PHONEMeS: efficient modeling of signaling networks derived from large‐scale mass spectrometry data. J Proteome Res
20: 2138–2144
3368241610.1021/acs.jproteome.0c00958

Hass
H
, 
Loos
C
, 
Raimúndez‐Álvarez
E
, 
Timmer
J
, 
Hasenauer
J
, 
Kreutz
C
 (2019) Benchmark problems for dynamic modeling of intracellular processes. Bioinformatics
35: 3073–3082
3062460810.1093/bioinformatics/btz020PMC6735869

Haynes
WA
, 
Higdon
R
, 
Stanberry
L
, 
Collins
D
, 
Kolker
E
 (2013) Differential expression analysis for pathways. PLoS Comput Biol
9: e1002967
2351635010.1371/journal.pcbi.1002967PMC3597535

Haynes
WA
, 
Tomczak
A
, 
Khatri
P
 (2018) Gene annotation bias impedes biomedical research. Sci Rep
8: 1362
2935874510.1038/s41598-018-19333-xPMC5778030

Hidalgo
MR
, 
Cubuk
C
, 
Amadoz
A
, 
Salavert
F
, 
Carbonell‐Caballero
J
, 
Dopazo
J
 (2017) High throughput estimation of functional cell activities reveals disease mechanisms and predicts relevant clinical outcomes. Oncotarget
8: 5160–5178
2804295910.18632/oncotarget.14107PMC5354899

Hornbeck
PV
, 
Zhang
B
, 
Murray
B
, 
Kornhauser
JM
, 
Latham
V
, 
Skrzypek
E
 (2015) PhosphoSitePlus, 2014: mutations, PTMs and recalibrations. Nucleic Acids Res
43: D512–D520
2551492610.1093/nar/gku1267PMC4383998

Hosseini‐Gerami
L
, 
Collier
DA
, 
Laing
E
, 
Evans
D
, 
Broughton
H
, 
Bender
A
 (2022) Benchmarking causal reasoning algorithms for gene expression‐based compound mechanism of action analysis. Res Sq
10.21203/rs.3.rs-1239049/v1
PMC1011179237072707

Hrovatin
K
, 
Fischer
DS
, 
Theis
FJ
 (2022) Toward modeling metabolic state from single‐cell transcriptomics. Mol Metab
57: 101396
3478539410.1016/j.molmet.2021.101396PMC8829761

Ihnatova
I
, 
Popovici
V
, 
Budinska
E
 (2018) A critical comparison of topology‐based pathway analysis methods. PLoS One
13: e0191154
2937022610.1371/journal.pone.0191154PMC5784953

Jacob
L
, 
Neuvial
P
, 
Dudoit
S
 (2012) More power via graph‐structured tests for differential expression of gene networks. Ann Appl Stat
6: 561–600


Judeh
T
, 
Johnson
C
, 
Kumar
A
, 
Zhu
D
 (2013) TEAK: topology enrichment analysis framework for detecting activated biological subpathways. Nucleic Acids Res
41: 1425–1437
2326844810.1093/nar/gks1299PMC3561980

Kanehisa
M
, 
Furumichi
M
, 
Sato
Y
, 
Ishiguro‐Watanabe
M
, 
Tanabe
M
 (2021) KEGG: integrating viruses and cellular organisms. Nucleic Acids Res
49: D545–D551
3312508110.1093/nar/gkaa970PMC7779016

Köksal
AS
, 
Beck
K
, 
Cronin
DR
, 
McKenna
A
, 
Camp
ND
, 
Srivastava
S
, 
ME
MG
, 
Bodík
R
, 
Wolf‐Yadlin
A
, 
Fraenkel
E

*et al* (2018) Synthesizing Signaling pathways from temporal phosphoproteomic data. Cell Rep
24: 3607–3618
3025721910.1016/j.celrep.2018.08.085PMC6295338

Koleti
A
, 
Terryn
R
, 
Stathias
V
, 
Chung
C
, 
Cooper
DJ
, 
Turner
JP
, 
Vidovic
D
, 
Forlin
M
, 
Kelley
TT
, 
D'Urso
A

*et al* (2018) Data Portal for the Library of Integrated Network‐based Cellular Signatures (LINCS) program: integrated access to diverse large‐scale cellular perturbation response data. Nucleic Acids Res
46: D558–D566
2914046210.1093/nar/gkx1063PMC5753343

Kustatscher
G
, 
Collins
T
, 
Gingras
A‐C
, 
Guo
T
, 
Hermjakob
H
, 
Ideker
T
, 
Lilley
KS
, 
Lundberg
E
, 
Marcotte
EM
, 
Ralser
M

*et al* (2022) Understudied proteins: opportunities and challenges for functional proteomics. Nat Methods
19: 774–779
3553463310.1038/s41592-022-01454-x

Le Novère
N
, 
Hucka
M
, 
Mi
H
, 
Moodie
S
, 
Schreiber
F
, 
Sorokin
A
, 
Demir
E
, 
Wegner
K
, 
Aladjem
MI
, 
Wimalaratne
SM

*et al* (2009) The systems biology graphical notation. Nat Biotechnol
27: 735–741
1966818310.1038/nbt.1558

Li
X
, 
Shen
L
, 
Shang
X
, 
Liu
W
 (2015) Subpathway analysis based on signaling‐pathway impact analysis of signaling pathway. PLoS One
10: e0132813
2620791910.1371/journal.pone.0132813PMC4514860

Licata
L
, 
Lo Surdo
P
, 
Iannuccelli
M
, 
Palma
A
, 
Micarelli
E
, 
Perfetto
L
, 
Peluso
D
, 
Calderone
A
, 
Castagnoli
L
, 
Cesareni
G
 (2020) SIGNOR 2.0, the SIGnaling network open resource 2.0: 2019 update. Nucleic Acids Res
48: D504–D510
3166552010.1093/nar/gkz949PMC7145695

Linding
R
, 
Jensen
LJ
, 
Pasculescu
A
, 
Olhovsky
M
, 
Colwill
K
, 
Bork
P
, 
Yaffe
MB
, 
Pawson
T
 (2008) NetworKIN: a resource for exploring cellular phosphorylation networks. Nucleic Acids Res
36: D695–D699
1798184110.1093/nar/gkm902PMC2238868

Liu
Y
, 
Beyer
A
, 
Aebersold
R
 (2016) On the dependency of cellular protein levels on mRNA abundance. Cell
165: 535–550
2710497710.1016/j.cell.2016.03.014

Liu
A
, 
Trairatphisan
P
, 
Gjerga
E
, 
Didangelos
A
, 
Barratt
J
, 
Saez‐Rodriguez
J
 (2019) From expression footprints to causal pathways: contextualizing large signaling networks with CARNIVAL. NPJ Syst Biol Appl
5: 40
3172820410.1038/s41540-019-0118-zPMC6848167

Lowe
R
, 
Shirley
N
, 
Bleackley
M
, 
Dolan
S
, 
Shafee
T
 (2017) Transcriptomics technologies. PLoS Comput Biol
13: e1005457
2854514610.1371/journal.pcbi.1005457PMC5436640

de Magalhães
JP
 (2021) Every gene can (and possibly will) be associated with cancer. Trends Genet
38: 216–217
3475647210.1016/j.tig.2021.09.005

Martens
M
, 
Ammar
A
, 
Riutta
A
, 
Waagmeester
A
, 
Slenter
DN
, 
Hanspers
K
, 
A Miller
R
, 
Digles
D
, 
Lopes
EN
, 
Ehrhart
F

*et al* (2021) WikiPathways: connecting communities. Nucleic Acids Res
49: D613–D621
3321185110.1093/nar/gkaa1024PMC7779061

Martini
P
, 
Sales
G
, 
Massa
MS
, 
Chiogna
M
, 
Romualdi
C
 (2013) Along signal paths: an empirical gene set approach exploiting pathway topology. Nucleic Acids Res
41: e19
2300213910.1093/nar/gks866PMC3592432

Massa
MS
, 
Chiogna
M
, 
Romualdi
C
 (2010) Gene set analysis exploiting the topology of a pathway. BMC Syst Biol
4: 121
2080993110.1186/1752-0509-4-121PMC2945950

McClendon
CL
, 
Kornev
AP
, 
Gilson
MK
, 
Taylor
SS
 (2014) Dynamic architecture of a protein kinase. Proc Natl Acad Sci U S A
111: E4623–E4631
2531926110.1073/pnas.1418402111PMC4217441

Needham
EJ
, 
Parker
BL
, 
Burykin
T
, 
James
DE
, 
Humphrey
SJ
 (2019) Illuminating the dark phosphoproteome. Sci Signal
12: eaau8645
3067063510.1126/scisignal.aau8645

Nilsson
A
, 
Peters
JM
, 
Bryson
B
 & 
Lauffenburger
DA
 (2021) Artificial neural networks enable genome‐scale simulations of intracellular signaling. *bioRxiv*
10.1101/2021.09.24.461703 [PREPRINT]PMC916307235654811

Ochoa
D
, 
Jarnuczak
AF
, 
Viéitez
C
, 
Gehre
M
, 
Soucheray
M
, 
Mateus
A
, 
Kleefeldt
AA
, 
Hill
A
, 
Garcia‐Alonso
L
, 
Stein
F

*et al* (2020) The functional landscape of the human phosphoproteome. Nat Biotechnol
38: 365–373
3181926010.1038/s41587-019-0344-3PMC7100915

Oughtred
R
, 
Rust
J
, 
Chang
C
, 
Breitkreutz
B‐J
, 
Stark
C
, 
Willems
A
, 
Boucher
L
, 
Leung
G
, 
Kolas
N
, 
Zhang
F

*et al* (2021) The BioGRID database: a comprehensive biomedical resource of curated protein, genetic, and chemical interactions. Protein Sci
30: 187–200
3307038910.1002/pro.3978PMC7737760

Page
L
, 
Brin
S
, 
Motwani
R
 & 
Winograd
T
 (1999) 
*The PageRank citation ranking bringing order to the web*. Stanford InfoLab


Paull
EO
, 
Carlin
DE
, 
Niepel
M
, 
Sorger
PK
, 
Haussler
D
, 
Stuart
JM
 (2013) Discovering causal pathways linking genomic events to transcriptional states using tied diffusion through interacting events (TieDIE). Bioinformatics
29: 2757–2764
2398656610.1093/bioinformatics/btt471PMC3799471

Perkel
JM
 (2021) Single‐cell proteomics takes Centre stage. Nature
597: 580–582
3454522510.1038/d41586-021-02530-6

Picelli
S
, 
Faridani
OR
, 
Björklund
AK
, 
Winberg
G
, 
Sagasser
S
, 
Sandberg
R
 (2014) Full‐length RNA‐seq from single cells using smart‐seq2. Nat Protoc
9: 171–181
2438514710.1038/nprot.2014.006

Potel
CM
, 
Kurzawa
N
, 
Becher
I
, 
Typas
A
, 
Mateus
A
, 
Savitski
MM
 (2021) Impact of phosphorylation on thermal stability of proteins. Nat Methods
18: 757–759
3414070010.1038/s41592-021-01177-5

Rian
K
, 
Hidalgo
MR
, 
Çubuk
C
, 
Falco
MM
, 
Loucera
C
, 
Esteban‐Medina
M
, 
Alamo‐Alvarez
I
, 
Peña‐Chilet
M
, 
Dopazo
J
 (2021) Genome‐scale mechanistic modeling of signaling pathways made easy: a bioconductor/cytoscape/web server framework for the analysis of omic data. Comput Struct Biotechnol J
19: 2968–2978
3413609610.1016/j.csbj.2021.05.022PMC8170118

Rodchenkov
I
, 
Babur
O
, 
Luna
A
, 
Aksoy
BA
, 
Wong
JV
, 
Fong
D
, 
Franz
M
, 
Siper
MC
, 
Cheung
M
, 
Wrana
M

*et al* (2020) Pathway Commons 2019 Update: integration, analysis and exploration of pathway data. Nucleic Acids Res
48: D489–D497
3164709910.1093/nar/gkz946PMC7145667

Saez‐Rodriguez
J
, 
Blüthgen
N
 (2020) Personalized signaling models for personalized treatments. Mol Syst Biol
16: e9042
3212994210.15252/msb.20199042PMC6951359

Satpathy
S
, 
Jaehnig
EJ
, 
Krug
K
, 
Kim
B‐J
, 
Saltzman
AB
, 
Chan
DW
, 
Holloway
KR
, 
Anurag
M
, 
Huang
C
, 
Singh
P

*et al* (2020) Microscaled proteogenomic methods for precision oncology. Nat Commun
11: 532
3198829010.1038/s41467-020-14381-2PMC6985126

Savitski
MM
, 
FBM
R
, 
Franken
H
, 
Werner
T
, 
Savitski
MF
, 
Eberhard
D
, 
Martinez Molina
D
, 
Jafari
R
, 
Dovega
RB
, 
Klaeger
S

*et al* (2014) Tracking cancer drugs in living cells by thermal profiling of the proteome. Science
346: 1255784
2527861610.1126/science.1255784

Schubert
M
, 
Klinger
B
, 
Klünemann
M
, 
Sieber
A
, 
Uhlitz
F
, 
Sauer
S
, 
Garnett
MJ
, 
Blüthgen
N
, 
Saez‐Rodriguez
J
 (2018) Perturbation‐response genes reveal signaling footprints in cancer gene expression. Nat Commun
9: 20
2929599510.1038/s41467-017-02391-6PMC5750219

Sobsey
CA
, 
Ibrahim
S
, 
Richard
VR
, 
Gaspar
V
, 
Mitsa
G
, 
Lacasse
V
, 
Zahedi
RP
, 
Batist
G
, 
Borchers
CH
 (2020) Targeted and untargeted proteomics approaches in biomarker development. Proteomics
20: e1900029
3172913510.1002/pmic.201900029

Stark
R
, 
Grzelak
M
, 
Hadfield
J
 (2019) RNA sequencing: the teenage years. Nat Rev Genet
20: 631–656
3134126910.1038/s41576-019-0150-2

Szalai
B
, 
Saez‐Rodriguez
J
 (2020) Why do pathway methods work better than they should?
FEBS Lett
594: 4189–4200
3327091010.1002/1873-3468.14011

Szklarczyk
D
, 
Gable
AL
, 
Nastou
KC
, 
Lyon
D
, 
Kirsch
R
, 
Pyysalo
S
, 
Doncheva
NT
, 
Legeay
M
, 
Fang
T
, 
Bork
P

*et al* (2021) The STRING database in 2021: customizable protein‐protein networks, and functional characterization of user‐uploaded gene/measurement sets. Nucleic Acids Res
49: D605–D612
3323731110.1093/nar/gkaa1074PMC7779004

Tang
F
, 
Barbacioru
C
, 
Wang
Y
, 
Nordman
E
, 
Lee
C
, 
Xu
N
, 
Wang
X
, 
Bodeau
J
, 
Tuch
BB
, 
Siddiqui
A

*et al* (2009) mRNA‐Seq whole‐transcriptome analysis of a single cell. Nat Methods
6: 377–382
1934998010.1038/nmeth.1315

Tarca
AL
, 
Draghici
S
, 
Khatri
P
, 
Hassan
SS
, 
Mittal
P
, 
Kim
J‐S
, 
Kim
CJ
, 
Kusanovic
JP
, 
Romero
R
 (2009) A novel signaling pathway impact analysis. Bioinformatics
25: 75–82
1899072210.1093/bioinformatics/btn577PMC2732297

Terfve
CDA
, 
Wilkes
EH
, 
Casado
P
, 
Cutillas
PR
, 
Saez‐Rodriguez
J
 (2015) Large‐scale models of signal propagation in human cells derived from discovery phosphoproteomic data. Nat Commun
6: 8033
2635468110.1038/ncomms9033PMC4579397

Touré
V
, 
Vercruysse
S
, 
Acencio
ML
, 
Lovering
RC
, 
Orchard
S
, 
Bradley
G
, 
Casals‐Casas
C
, 
Chaouiya
C
, 
Del‐Toro
N
, 
Flobak
Å

*et al* (2020) The minimum information about a molecular interaction causal statement (MI2CAST). Bioinformatics
36: 5712–5718
10.1093/bioinformatics/btaa622PMC802367432637990

Türei
D
, 
Korcsmáros
T
, 
Saez‐Rodriguez
J
 (2016) OmniPath: guidelines and gateway for literature‐curated signaling pathway resources. Nat Methods
13: 966–967
2789806010.1038/nmeth.4077

Vandin
F
, 
Clay
P
, 
Upfal
E
, 
Raphael
BJ
 (2012) Discovery of mutated subnetworks associated with clinical data in cancer. Pac Symp Biocomput: 55–66
22174262

Vanschoren
J
, 
van Rijn
JN
, 
Bischl
B
, 
Torgo
L
 (2014) OpenML. SIGKDD Explor Newsl
15: 49–60


Vogt
T
, 
Czauderna
T
, 
Schreiber
F
 (2013) Translation of SBGN maps: process description to activity flow. BMC Syst Biol
7: 115
2417608810.1186/1752-0509-7-115PMC4228393

Weber
LM
, 
Saelens
W
, 
Cannoodt
R
, 
Soneson
C
, 
Hapfelmeier
A
, 
Gardner
PP
, 
Boulesteix
A‐L
, 
Saeys
Y
, 
Robinson
MD
 (2019) Essential guidelines for computational method benchmarking. Genome Biol
20: 125
3122119410.1186/s13059-019-1738-8PMC6584985

Wei
C‐H
, 
Allot
A
, 
Leaman
R
, 
Lu
Z
 (2019) PubTator central: automated concept annotation for biomedical full text articles. Nucleic Acids Res
47: W587–W593
3111488710.1093/nar/gkz389PMC6602571

Weidemüller
P
, 
Kholmatov
M
, 
Petsalaki
E
, 
Zaugg
JB
 (2021) Transcription factors: bridge between cell signaling and gene regulation. Proteomics
21: e2000034
3431409810.1002/pmic.202000034

Wicherts
JM
, 
Veldkamp
CLS
, 
Augusteijn
HEM
, 
Bakker
M
, 
van Aert
RCM
, 
van Assen
MALM
 (2016) Degrees of freedom in planning, running, analyzing, and reporting psychological studies: a checklist to avoid p‐hacking. Front Psychol
7: 1832
2793301210.3389/fpsyg.2016.01832PMC5122713
